# Inhibition of IKKβ Reduces Ethanol Consumption in C57BL/6J Mice

**DOI:** 10.1523/ENEURO.0256-16.2016

**Published:** 2016-10-31

**Authors:** Jay M. Truitt, Yuri A. Blednov, Jillian M. Benavidez, Mendy Black, Olga Ponomareva, Jade Law, Morgan Merriman, Sami Horani, Kelly Jameson, Amy W. Lasek, R. Adron Harris, R. Dayne Mayfield

**Affiliations:** 1 Waggoner Center for Alcohol and Addiction Research, The University of Texas at Austin, Austin, Texas 78712; 2 Department of Psychiatry, University of Illinois at Chicago, Chicago, Illinois 60612

**Keywords:** alcohol, astrocytes, microglia, neurons, sulfasalazine, TPCA-1, Cre recombinase, nucleus accumbens, central amygdala, DID, binge drinking

## Abstract

Proinflammatory pathways in neuronal and non-neuronal cells are implicated in the acute and chronic effects of alcohol exposure in animal models and humans. The nuclear factor-κB (NF-κB) family of DNA transcription factors plays important roles in inflammatory diseases. The kinase IKKβ mediates the phosphorylation and subsequent proteasomal degradation of cytosolic protein inhibitors of NF-κB, leading to activation of NF-κB. The role of IKKβ as a potential regulator of excessive alcohol drinking had not previously been investigated. Based on previous findings that the overactivation of innate immune/inflammatory signaling promotes ethanol consumption, we hypothesized that inhibiting IKKβ would limit/decrease drinking by preventing the activation of NF-κB. We studied the systemic effects of two pharmacological inhibitors of IKKβ, TPCA-1 and sulfasalazine, on ethanol intake using continuous- and limited-access, two-bottle choice drinking tests in C57BL/6J mice. In both tests, TPCA-1 and sulfasalazine reduced ethanol intake and preference without changing total fluid intake or sweet taste preference. A virus expressing *Cre* recombinase was injected into the nucleus accumbens and central amygdala to selectively knock down IKKβ in mice genetically engineered with a conditional *Ikkb* deletion (*Ikkb^F/F^*). Although IKKβ was inhibited to some extent in astrocytes and microglia, neurons were a primary cellular target. Deletion of IKKβ in either brain region reduced ethanol intake and preference in the continuous access two-bottle choice test without altering the preference for sucrose. Pharmacological and genetic inhibition of IKKβ decreased voluntary ethanol consumption, providing initial support for IKKβ as a potential therapeutic target for alcohol abuse.

## Significance Statement

Alcoholism is a devastating disease with few pharmacological treatment options. The disease pathophysiology is unknown, but it is increasingly evident that proinflammatory signaling plays a role. Nuclear factor-κB (NF-κB) is a transcription factor that controls the expression of genes that are involved in inflammation and immunity. IKKβ is a kinase that plays an essential role in regulating the NF-κB signaling pathway. The role of IKKβ in alcohol drinking had not previously been investigated. Our goal was to assess the peripheral and central effects of IKKβ on long-term and binge-like alcohol consumption, and its potential role as a therapeutic target to reduce drinking.

## Introduction

Alcohol exposure is known to activate peripheral and central proinflammatory pathways (Robinson et al., 2014; Crews and Vetreno, 2016). Genomic evidence for alcohol-induced inflammatory- and immune-related signaling comes from genetic association studies in alcoholic individuals (Pastor et al., 2000, 2005; Edenberg et al., 2008; Saiz et al., 2009), gene expression microarray studies from postmortem brains of alcoholic individuals (Liu et al., 2006; Ökvist et al., 2007), and transcriptome meta-analyses in selectively bred mice (Mulligan et al., 2006) and ethanol-exposed mice (Gorini et al., 2013a,b; Nunez et al., 2013; Osterndorff-Kahanek et al., 2013). Behavioral validation studies showed that mice with null mutations of different immune-related genes drank less ethanol (Blednov et al., 2012), while stimulation of innate immune responses using lipopolysaccharide produced prolonged increases in drinking (Blednov et al., 2011). Many of the inflammatory-related genes implicated in these studies mediate their effects through nuclear factor-κB (NF-κB).

NF-κB transcription family members are ubiquitously expressed throughout the body and play important roles in innate/adaptive immunity, cell survival, and inflammation (Scheidereit, 2006; Perkins, 2007). NF-κB transcriptional activity is regulated by inhibitory IκB proteins. The IκB kinase (IKK) complex mediates the phosphorylation and degradation of IκB, allowing translocation of active NF-κB to the nucleus, where it acts as a transcription factor for numerous proinflammatory chemokines/cytokines, such as TNF-α and IL-6 (Schmid and Birbach, 2008; Gamble et al., 2012). The IKK complex represents a point of convergence for many inflammatory extracellular signals, and plays a key role in inflammation and disease (Schmid and Birbach, 2008; Gamble et al., 2012). IKKβ specifically mediates the classical/canonical NF-κB pathway (Schmid and Birbach, 2008), has a clearly established role as an intermediate in NF-κB-induced cellular inflammation, and is involved in many inflammatory diseases (Grivennikov et al., 2010; Sunami et al., 2012).

Studies that have examined the effects of ethanol on IKKβ focused on peripheral effects, such as the exacerbation of pancreatic and hepatic inflammation by chronic ethanol (Sunami et al., 2012; Huang et al., 2015). Studies of the central actions of IKKβ have concentrated on neurodegenerative or metabolic disorders, but did not involve ethanol exposure (Zhang et al., 2008; Maqbool et al., 2013). Other studies have shown that IKKβ gene expression was altered in postmortem prefrontal cortex (PFC) from alcoholic individuals (Flatscher-Bader et al., 2005) and mouse PFC following ethanol exposure and in selectively bred animals predisposed to drink alcohol (Mulligan et al., 2006; Osterndorff-Kahanek et al., 2015). To date, no studies have explored the peripheral or central effects of IKKβ on ethanol drinking. IKKβ is a compelling target for study, given its role in inflammatory diseases, and its role in mediating cocaine sensitization and reward through plasticity-dependent neuronal signaling in the nucleus accumbens (NAc; Russo et al., 2009). Furthermore, IKKβ mediated the prodepressant and anxiogenic effects of chronic stress through neuronal plasticity mechanisms in the NAc (Christoffel et al., 2011, 2012).

We examined different methods (pharmacological and genetic) to inhibit IKKβ, different brain regions/cell types, and different two-bottle choice (2BC) ethanol drinking paradigms in mice. Two different peripherally acting IKKβ inhibitors, TPCA-1 (2-[(aminocarbonyl) amino]-5-(4-fluorophenyl)-3-thiophenecarboxamide) and sulfasalazine, were tested in long-term and binge-like drinking models. TPCA-1 is a selective small-molecule inhibitor of IKKβ (Podolin et al., 2005). Sulfasalazine does not cross the blood–brain barrier (BBB; Liu et al., 2012); possesses strong IKKβ inhibitory activity; and is used to treat inflammatory bowel disease, ulcerative colitis, and Crohn’s disease (Lappas et al., 2005). We then examined the effects of *Cre*-mediated IKKβ knockdown in different cell types in the NAc or central amygdala (CeA) on voluntary ethanol consumption. Based on previous studies (Blednov et al., 2011, 2012), we hypothesized that the inhibition of IKKβ would decrease proinflammatory signaling and reduce alcohol drinking.

## Materials and Methods

### Animals

Pharmacological antagonist studies were conducted in adult male C57BL/6J mice (original breeders were purchased from The Jackson Laboratory). Genetic knock-down studies were performed in adult male mice with a floxed *Ikkb* gene on a C57BL/6J background (i.e., C57BL/6J mice with *Ikkb* flanked by LoxP sites, denoted as *Ikkb^F/F^*). Original breeders were acquired from Casey W. Wright (College of Pharmacy, The University of Texas at Austin, Austin, TX). The C57BL/6J strain was chosen because of its propensity for voluntary ethanol consumption (Belknap et al., 1997). Mice were group housed four or five per cage on a 12 h light/dark cycle (lights on at 7:00 A.M.) with *ad libitum* access to water and rodent chow (Prolab RMH 180 5LL2 chow, TestDiet) in temperature- and humidity-controlled rooms. Behavioral testing began when the mice were at least 2 months old. Mice were individually housed at least 2 weeks before beginning the drinking tests. Experiments were conducted in isolated behavioral testing rooms in the Animal Resources Center at The University of Texas at Austin. All experiments were approved by The University of Texas Institutional Animal Care and Use Committee and were conducted in accordance with National Institutes of Health guidelines with regard to the use of animals in research.

### Pharmacological inhibitors of IKKβ

Sulfasalazine (Sigma-Aldrich) was injected intraperitoneally, and TPCA-1 (Tocris Bioscience) was administered by mouth. Both drugs were freshly prepared as suspensions in saline solution, with four to five drops of Tween-80, and were injected in a volume of 0.1 ml/10 g of body weight for intraperitoneal administration, and 0.05 ml/10 g of body weight for oral administration. Drugs were administered 30 min prior to ethanol presentation times (see below). Doses of drugs and routes of administration were based on published data that showed anti-inflammatory activity *in vivo*.

### Brain region-specific lentiviral-mediated knockdown of IKKβ

*Ikkb^F/F^* mice were injected bilaterally (into the NAc or CeA) with either a vesicular stomatitis virus glycoprotein (VSV-G) pseudotyped lentivirus (LV) expressing *Cre* recombinase fused to enhanced green fluorescent protein (EGFP) under the control of a cytomegalovirus (CMV) promoter (LV-Cre-EGFP) or an “empty” VSV-G pseudotyped lentiviral vector expressing only the EGFP transgene under a CMV promoter. Mice were anesthetized by isoflurane inhalation, were placed in a stereotaxic apparatus (model 1900, David Kopf Instruments), and were administered a preoperative analgesic (Rimadyl 5 mg/kg). The skull was exposed, and bregma and lambda were visualized with a dissecting microscope. A digitizer attached to the micromanipulator of the stereotaxic apparatus was used to locate coordinates relative to bregma. Burr holes were drilled bilaterally above the injection sites in the skull using a drill equipped with a #75 carbide bit (David Kopf Instruments). The injection sites targeted either the NAc [using the following coordinates relative to bregma: anteroposterior (AP) +1.49 mm, mediolateral (ML) ±0.9 mm, dorsoventral (DV) −4.8 mm] or the CeA (using the following coordinates: AP −1.14 mm, ML ±2.84 mm, DV −4.8 mm). Injections were performed using a 10 μl microsyringe (model #1701, Hamilton) and a 30 gauge needle. The needle of the syringe was lowered to the DV coordinate and retracted 0.2 mm. Virus solutions (1.0 μl with a titer of 1.8 × 10^8^ viral particles/ml in PBS) were injected into each site at a rate of 200 nl/min. After each injection, the syringe was left in place for 5 min before being retracted over a period of 3 min. Incisions were closed with tissue adhesive (Vetbond, 3M). Mice were individually housed after surgery and given a 4 week recovery period before starting the ethanol drinking tests.

### Behavioral testing

The following three different ethanol-drinking models were used in this study: (1) continuous 24 h 2BC with access to water and ethanol (15%, v/v); (2) 2BC drinking-in-the-dark (DID) with limited 3 h access to 15% ethanol (2BC-DID); and (3) 2BC using ascending concentrations of ethanol solutions (3–16%; see below).

#### Pharmacological inhibitors of IKKβ

The effects IKKβ antagonists on ethanol intake were measured in adult male C57BL/6J mice in two different drinking paradigms: 2BC with 15% ethanol and 2BC-DID per the protocols previously described (Blednov et al., 2003, 2014). For both tests, mice were pretrained to consume 15% ethanol for at least 3 weeks to provide stable consumption. Ethanol intake was measured after saline injection (intraperitoneally or by mouth, corresponding to the route of administration for the antagonists) for 2 d, and mice were grouped to provide similar levels of ethanol intake and preference. In the 2BC test, measurements of ethanol intake were made 6 and 24 h after beginning the drinking test, which began immediately after lights off. In the 2BC-DID test, drinking began 3 h after lights off and lasted for 3 h. Ethanol intake was measured once at the end of the 3 h drinking period. The position of the drinking tubes was changed daily to control for side preferences. Mice were weighed every 4 d. For both experiments, ethanol consumption (in grams per kilogram), preference (ratio of alcohol consumption to total fluid consumption), and total fluid intake (in grams per kilogram) were measured at the appropriate time points.

#### Brain region-specific lentiviral-mediated knockdown of IKKβ

The effects of IKKβ knockdown in the NAc or CeA on ethanol consumption were measured in adult male *Ikkb^F/F^* mice using the 24 h 2BC test. Mice treated with either LV-Cre-EGFP or LV-EGFP-Empty were given continuous access to water and ascending concentrations of ethanol solutions (3%, 6%, 8%, 10%, 12%, 14%, and 16%, v/v) at 2 d intervals (Blednov et al., 2014). The position of administration tubes was changed daily to control for position preferences. Mice were weighed every 4 d.

#### Preference for saccharin

One month after completion of the 2BC ethanol test described above, *Ikkb^F/F^* mice were tested for saccharin preference using the 2BC protocol. Mice were offered saccharin in increasing concentrations (0.008%, 0.016%, and 0.033%), and 24 h intake was calculated. Each concentration was offered for 2 d, and bottle positions were changed daily. The low concentration was presented first, followed by the higher concentrations.

### RNA isolation

After the completion of behavioral testing, mice were killed by cervical dislocation and decapitated. The brains were quickly removed, flash frozen in liquid nitrogen, and later embedded in Optimal Cutting Temperature (OCT) media in isopentane on dry ice. Brains were then stored at −80°C for future processing. Brains were transferred to a cryostat set at −6°C for at least 1 h before sectioning. Sections (300 μm) were collected from +1.80 to +0.60 mm (AP) relative to bregma and transferred to precooled glass slides on dry ice. Micropunch sampling was performed on a frozen stage (−25°C) using Dual Fluorescent Protein Flashlight (Nightsea), and a mouse stereotaxic atlas to identify the GFP expression and anatomical location of the injection site. Microdissection punches (Stoelting Co.) with an inner diameter of 0.75 mm were used to obtain samples of NAc. This inner diameter fit within the viral spread around the injection site and minimized contamination from other tissue. Punches were taken bilaterally from 4–300 μm sections and were stored at −80°C until RNA extraction. Micropunches were washed with 100% ethanol and RNaseZap (Life Technologies) between each animal. All equipment used to obtain tissue was treated with RNaseZap (Life Technologies) to prevent RNA degradation. Total RNA was extracted using the MagMAX-96 for Microarrays Total RNA Isolation Kit (Life Technologies) according to the manufacturer instructions. RNA yields and purity were assessed using a NanoDrop 8000 spectrophotometer (Thermo Fisher Scientific). Both the 260:230 and 260:280 ratios were >2.0. RNA quality was determined using the Agilent 2200 TapeStation (Agilent) with RNA integrity numbers averaging >8.0.

### Quantitative PCR

To verify *Ikkb* mRNA knockdown, single-stranded cDNA was synthesized from total RNA using the TaqMan High Capacity RNA-to-cDNA Kit (Life Technologies). Following reverse transcription, quantitative real-time PCR was performed in triplicate using TaqMan Gene Expression Assays together with the TaqMan Gene Expression Master Mix (Life Technologies), per the manufacturer instructions. The TaqMan Gene Expression Assays used were *Ikbkb* (ID #Mm01222247_m1), *Tnfa* (ID #Mm00443258_m1), *Il6* (ID #Mm00446190_m1), and EGFP (ID #Mr04097229_mr). *Gapdh* (Mm99999915_g1; glyceraldehyde-3-phosphate dehydrogenase) was used as a reference gene, and relative mRNA levels were determined using the 2^−▵▵CT^ method (Schmittgen and Livak, 2008). *Gapdh* was used as the endogenous control because of its low variability between samples. Reactions were performed in a CFX384 Real-Time PCR Detection System (Bio-Rad), and data were collected using CFX Manger (Bio-Rad). All genes were normalized to the endogenous housekeeping gene *Gapdh* and expressed relative to the respective LV-EGFP-Empty control treatment.

### Immunohistochemistry

#### Tissue harvesting

Animals were killed, transcardially perfused with PBS and 4% paraformaldehyde (PFA); and brains were harvested, postfixed for 24 h in 4% PFA at 4°C, and cryoprotected for 24 h in 20% sucrose in PBS at 4°C. Brains were placed in molds containing OCT compound (VWR) and frozen in isopentane on dry ice. The brains were equilibrated in a −12 to −14°C cryostat (Thermo Fisher Scientific) for at least 1 h, and coronal sections of 30 µm were taken from the NAc and CeA and placed in sterile PBS.

#### Immunostaining

Sections were penetrated with 0.1% Triton X-100 (2 × 10 min at 25°C); washed in PBS (3 × 5 min at 25°C); blocked with 10% goat or donkey serum (30 min at 25°C); treated with 1:250 anti-IKKβ (Millipore), 1:500 anti-NeuN (Santa Cruz Biotechnology), 1:300 anti-GFAP (Santa Cruz Biotechnology), 1:1000 anti-IBA1 (Dako), and 1:1000 anti-GFP (Santa Cruz Biotechnology) antibodies (4°C overnight); washed in PBS (3 × 10 min at 25°C); and then subjected to reaction with fluorescence-conjugated secondary antibodies of 1:1000 Alexa Fluor 488 and 1:1000 Alexa Fluor 568 (Invitrogen; 2 h at 25°C); and rinsed with PBS (3 × 10 min at 25°C). The sections were mounted on slides using sterile 0.2% gelatin and DAPI mounting media (Vector Laboratories) and coverslipped. Images were taken using either an Axiovert 200M Fluorescent Microscope (Zeiss) equipped with a 20× objective or an LSM 710 Confocal Microscope (Zeiss) equipped with a 63× objective. For the immunohistochemistry, the following two sets of control experiments were performed to test specificity: (1) replacement of the primary antibody with only the serum of the appropriate species; and (2) omission of secondary antibodies. No immunostaining was detected under either of these conditions.

#### Target verification

Serial sections (30 µm) of NAc (AP, +2.00 to 0.00 mm) and CeA (AP 0.00 to −2.00 mm) were mounted on slides with DAPI mounting media (Vector Laboratories) and visualized using an Axiovert 200M Fluorescent Microscope (Zeiss) equipped with a 10× objective to assess the location of the injection site. The quality of injection was quantitatively scored based of the strength of EGFP viral expression, injection location relative to target, and the spread of the virus. The injection was considered to be on target if the needle placement was within 0.3 mm of the desired stereotaxic coordinates and the virus EGFP expression covered at least one-third of the brain region of interest (i.e., NAc and CeA) on at least one side of the brain.

#### Image analysis

Brain sections were prepared as described in the Immunohistochemistry subsection. Epifluorescent images were acquired using an Axiovert 200M Fluorescent Microscope (Zeiss) equipped with a 20× objective and an automated stage. Images of the brain region of interest were captured (multiple 20× images in red, green, and blue channels) then were stitched together creating a composite view for further analysis. Images were taken without saturating the signal and digitized at 8 bits using the full intensity range of 0–256, and imported into the ImageJ software package (http://imagej.nih.gov/ij/). Composite images were split into individual channels and overlaid with a grid, and colocalized cells were counted. An LSM 710 Confocal Microscope (Zeiss) equipped with a 63× objective was used to take representative images for IKKβ cell-type specificity viral trophism.

### Statistical analysis

Numerical data are shown as the mean ± SEM, and *n* represents the number of animals tested. Data were analyzed using either ANOVA with repeated measures followed by Bonferroni *post hoc* tests or Student’s *t* tests, as appropriate (GraphPad Software). Calculated *p* values <0.05 were considered to be statistically significant.

## Results

### Pharmacological inhibitors of IKKβ reduce ethanol consumption and preference in the continuous 24 h 2BC test

**Figure 1.**
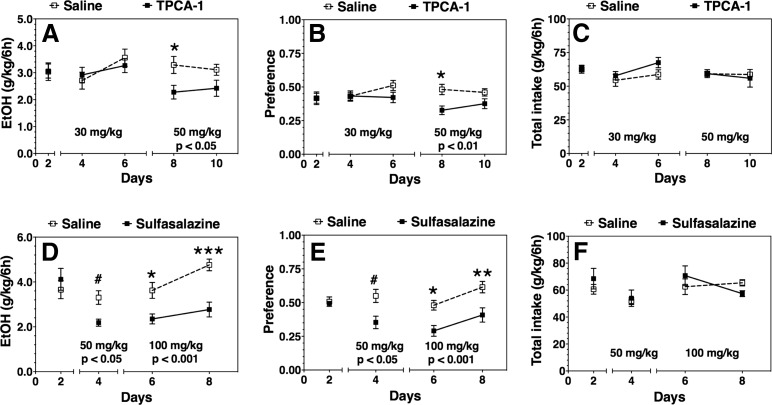
Effect of systemic administration of IKKβ inhibitors on ethanol (EtOH) intake and preference after 6 h of a continuous 24 h two-bottle choice test in C57BL/6J mice. ***A–C***, TPCA-1 (30 and 50 mg/kg) vs saline treated (*n* = 13 per group). ***D–F***, Sulfasalazine (50 and 100 mg/kg) vs saline treated (*n* = 6 per group). ***A***, ***D***, 15% ethanol consumption (g/kg/6 h). ***B***, ***E***, Preference for ethanol. ***C***, ***F***, Total fluid intake (g/kg/6 h). Day 2 in each panel shows the averages of 2 d of saline injections for each group ± SEM. Remaining time points are the 2 d drinking averages in the presence of saline or drug ± SEM. Significant main effect of drug treatment is shown by the *p* value beneath the treatment dose (two-way ANOVA with repeated measures). Significant *post hoc* effect of each drug compared with the corresponding saline group is indicated by the symbols above each time point (Bonferroni test for multiple comparisons, *p < 0.05, **p < 0.01, ***p < 0.001; or Student’s *t* test, #*p* < 0.05).

We first investigated the effects of systemic IKKβ inhibition on voluntary ethanol drinking. A pharmacological approach was selected because IKKβ genetic deletion causes embryonic lethality due to liver degeneration and apoptosis (Tanaka et al., 1999). Low and high doses of TPCA-1 or sulfasalazine were administered to adult male C57BL/6J mice on a daily basis. Voluntary ethanol (15%) drinking was evaluated using a continuous 24-h 2BC test. The lower dose of TPCA-1 (30 mg/kg) did not significantly alter ethanol intake, but the higher dose (50 mg/kg) reduced ethanol intake (*F*_(1,18)_ = 6.9, *p* < 0.05) and preference (*F*_(1,18)_ = 8.3, *p* < 0.01) 6 h after administration (Fig. 1*A*,*B*). Both doses of sulfasalazine reduced ethanol intake (Student’s *t* test, *p* < 0.05 and *F*_(1,10)_ = 24.1, *p* < 0.001) and preference (Student’s *t* test, *p* < 0.05 and *F*_(1,10)_ = 12.4, *p* < 0.01; Fig. 1*D*,*E*). No changes in total fluid intake were observed after the administration of either drug (Fig. 1*C*,*F*). There were no differences in ethanol intake or preference between drug- and saline-treated groups 18 h post-treatment for either drug (data not shown).

### Pharmacological inhibitors of IKKβ reduce ethanol consumption and preference in the limited-access drinking-in-the-dark 2BC test

**Figure 2.**
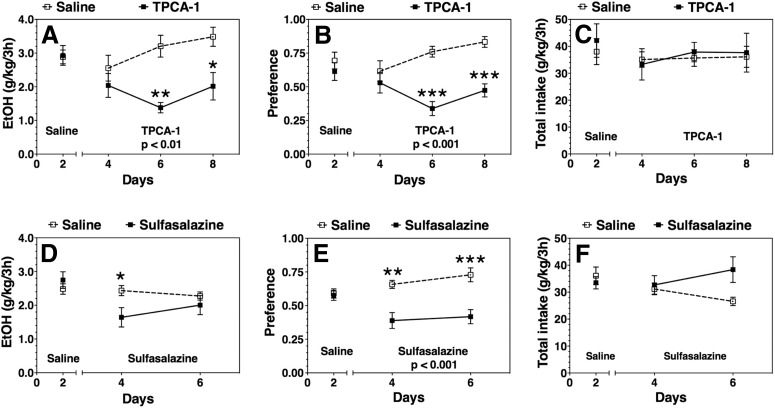
Effect of IKKβ inhibitors on ethanol (EtOH) intake and preference after 3 h in a limited access two-bottle choice drinking-in-the-dark test in C57BL/6J mice. ***A–C***, 50 mg/kg TPCA-1 vs saline treated (*n* = 6 per group). ***D–F***, 100 mg/kg sulfasalazine vs saline treated (*n* = 8 per group). ***A***, ***D***, 15% ethanol consumption (g/kg/3 h). ***B***, ***E***, Preference for ethanol. ***C***, ***F***, Total fluid intake (g/kg/3 h). Day 2 in each panel shows the averages of 2 d of saline injections for each group ± SEM. Remaining time points are the 2 d drinking averages in the presence of saline or drug ± SEM. Significant main effect of drug treatment is shown by the *p* value (two-way ANOVA with repeated measures). Significant *post hoc* effect of each drug compared with the corresponding saline group is indicated by the symbols above each time point (Bonferroni test for multiple comparisons, *p < 0.05, **p < 0.01, ***p < 0.001).

We administered TPCA-1 (50 mg/kg) or sulfasalazine (100 mg/kg) daily to a different cohort of adult male C57BL/6J mice and performed a 2BC test with limited 3 h access to 15% ethanol during the dark phase of the light/dark cycle, referred to as the 2BC-DID test. Compared with the continuous 2BC test, the 2BC-DID paradigm more closely replicates binge drinking, where mice typically consume higher levels of ethanol and exhibit behavioral evidence of intoxication (Thiele and Navarro, 2014). In this model, TPCA-1 reduced ethanol consumption (*F*_(1,10)_ = 14.0, *p* < 0.01) and preference (*F*_(1,10)_ = 21.6, *p* < 0.01) without affecting total fluid intake (Fig. 2*A–C*). Sulfasalazine, however, did not significantly alter ethanol or total fluid intake, but did reduce ethanol preference (*F*_(1,14)_ = 31.7, *p* < 0.001; Fig. 2*D–F*). There was a significant interaction between treatment and the time of ethanol consumption with a gradual time-dependent decrease in the effect of sulfasalazine (Fig. 2*D*).

### Brain region-specific knockdown of IKKβ in the NAc or CeA reduces ethanol consumption and preference in the continuous 24 h 2BC test

**Figure 3.**
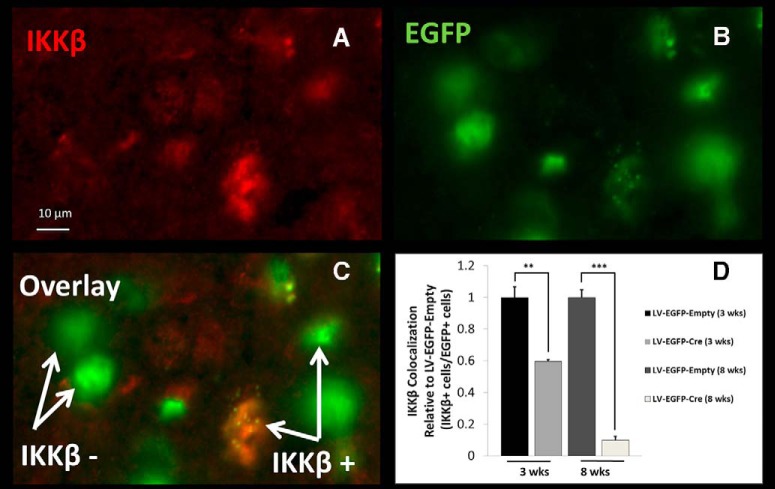
IKKβ protein knockdown (3 and 8 weeks post-injection) in NAc of *Ikkb^F/F^* mice. A fluorescent light microscope image of a representative stain from the 3 week post-injection time point in NAc is shown. ***A***, Anti-IKKβ fluorescently labeled antibody. ***B***, Anti-EGFP fluorescently labeled antibody. ***C***, Overlay of ***A*** and ***B*** (“IKKβ–” represents transduced cells without IKKβ, and “IKKβ+” represents transduced cells with IKKβ). ***D***, Knockdown of IKKβ (LV-EGFP-Cre) measured by IKKβ-positive cells colocalized with EGFP-positive cells relative to their time-matched control (LV-EGFP-Empty). The mean ± SEM of eight fields of view (20×) per mouse for four mice are shown (*n* = 4 for each group: 3 weeks after LV-EGFP-Cre, 3 weeks after LV-EGFP-Empty, 8 weeks after LV-EGFP-Cre, 8 weeks after LV-EGFP-Empty). Student’s *t* test: **p < 0.01, ***p < 0.001.

We next examined the role of IKKβ in two key areas of the brain implicated in the pathogenesis of alcohol use disorder (AUD). The NAc was chosen because it is part of the mesolimbic dopamine reward system that positively reinforces addictive behavior (Koob and Volkow, 2010; Koob, 2014). The NAc has also been implicated in IKKβ-mediated rewarding effects of cocaine **(** Russo et al., 2009**).** The CeA was selected because it is in involved in activating brain stress systems through the release of corticotropin-releasing factor and it negatively reinforces addictive behaviors (Koob and Le Moal, 2008; Koob and Volkow, 2010; Koob, 2014). Mice genetically engineered with a conditional *Ikkb* deletion (*Ikkb^F/F^*) were injected bilaterally in the brain region of interest with a lentivirus expressing either *Cre* fused to EGFP (LV-EGFP-Cre) or only EGFP (LV-EGFP-Empty). The transgenes of both viral vectors were under the control of a CMV promoter and were pseudotyped with VSV-G. Expression of *Cre* results in the excision of *Ikkb*. This method of targeted IKKβ deletion was validated by injecting LV-EGFP-Cre (*n* = 8) and LV-EGFP-Empty (*n* = 8) in the NAc of adult male *Ikkb^F/F^* mice followed by a 3- or 8-week incubation period. The time points were selected based on previous work in mouse brain showing maximal changes in expression 2 to 4 weeks post-injection (Ahmed et al., 2004). In addition, the 3- and 8-week post-injection time points were chosen to assess the level of IKKβ knockdown near the beginning (4 weeks post-injection) and end (8 weeks post-injection) of the drinking studies. At the appropriate time points, brains were perfused, harvested, sectioned, and immunostained with anti-IKKβ and anti-EGFP. The number of cells with the viral EGFP that colocalized with IKKβ were measured and compared between the LV-EGFP-Cre and LV-EGFP-Empty treatments at each time point. The relative expression of IKKβ in Cre-treated animals versus controls was 0.596 ± 0.012 (*p* < 0.01) at 3 weeks and 0.099 ± 0.023 (*p* < 0.001) at 8 weeks (Fig. 3). These represent a 40% and 90% decrease in IKKβ after 3 and 8 weeks, respectively.

**Figure 4.**
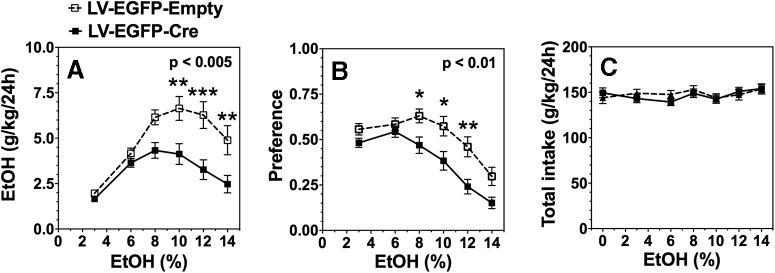
Effect of IKKβ knockdown in NAc on ethanol (EtOH) intake and preference during the 24 h two-bottle choice test in *Ikkb^F/F^* mice. ***A***, Ethanol consumption (g/kg/24 h). ***B***, Preference for ethanol. ***C***, Total fluid intake (g/kg/24 h). Each point is the average of 2 d of drinking ± SEM. Significant main effect of treatment is shown by the *p* value (two-way ANOVA with repeated measures). Significant *post hoc* effect of LV-EGFP-Cre compared with LV-EGFP-Empty treatment is indicated by symbols above each time point (Bonferroni test for multiple comparisons *p < 0.05, **p < 0.01). *n* = 32 animals injected with LV-Cre-EGFP; *n* = 20 injected with LV-Cre-Empty.

**Figure 5.**
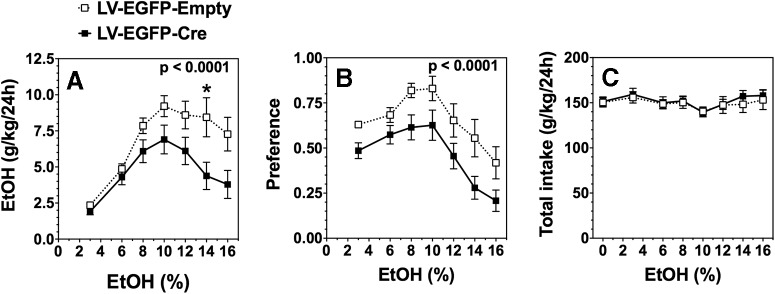
Effect of IKKβ knockdown in CeA on ethanol (EtOH) intake and preference during the 24 h two-bottle choice test in *Ikkb^F/F^* mice. ***A***, Ethanol consumption (g/kg/24 h). ***B***, Preference for ethanol. ***C***, Total fluid intake (g/kg/24 h). Significant main effect of treatment is shown by the *p* value (two-way ANOVA with repeated measures). Significant *post hoc* effect of LV-EGFP-Cre compared with LV-EGFP-Empty treatment is indicated by *p < 0.05 (Bonferroni test for multiple comparisons). *n* = 20 injected with LV-EGFP-Cre; *n* = 10 injected with LV-EGFP-Empty.

Subsequently, *Ikkb^F/F^* mice were injected bilaterally with LV-EGFP-Cre or LV-EGFP-Empty into either the NAc or CeA. After 4 weeks, the 2BC drinking test, in which mice could drink either water or a series of increasing ethanol concentrations ranging from 3% to 16%, was administered. Similar to the results after peripheral inhibition of IKKβ, targeted deletion of IKKβ in the NAc also reduced ethanol consumption (*F*_(1,50)_ = 10.0, *p* < 0.005) and preference (*F*_(1,50)_ = 8.3, *p* < 0.01) without affecting total fluid intake (Fig. 4*A–C*). Likewise, local deletion of IKKβ in the CeA reduced ethanol consumption (*F*_(1,196)_ = 19.1, *p* < 0.0001) and preference (*F*_(1,196)_ = 23.9, *p* < 0.0001) with no change in total fluid intake (Fig. 5*A–C*). At the higher ethanol concentrations, consumption and preference were reduced by >40% and 25%, respectively, after targeted knockdown in both regions (Figs. 4, 5).

**Figure 6.**
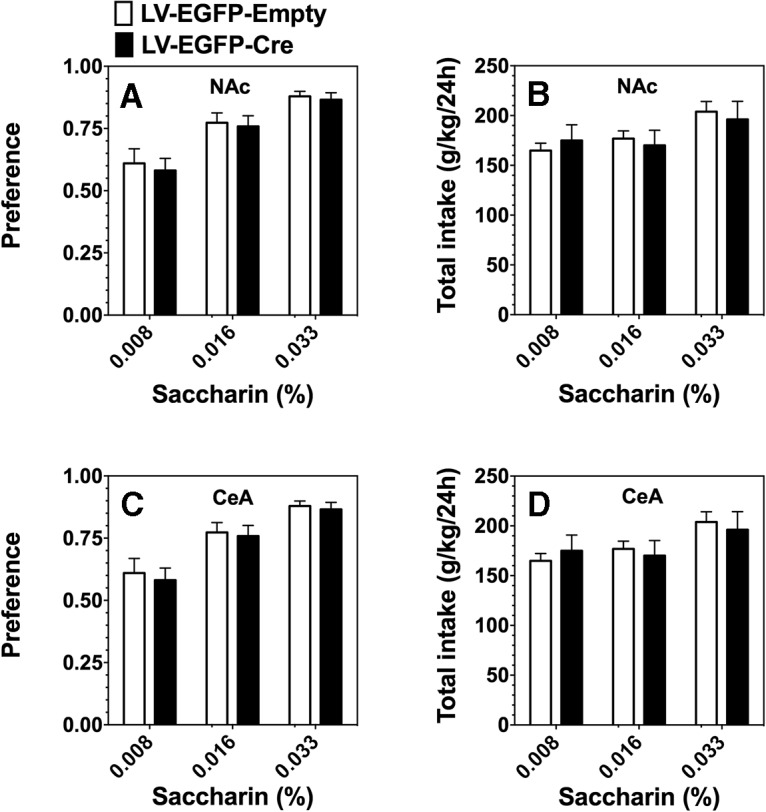
Lentiviral-mediated knockdown of IKKβ in the NAc and CeA had no effect on saccharin preference or total fluid intake in the 24 h two-bottle choice test in *Ikkb^F/F^* mice. ***A***, ***B***, The effect of IKKβ knockdown in NAc (*n* = 32, LV-EGFP-Cre; *n* = 20, LV-EGFP-Empty) is shown in ***A*** (preference for saccharin) and ***B*** (total fluid intake (g/kg/24 h). ***C***, ***D***, The effect of IKKβ knockdown in CeA (*n* = 20, LV-EGFP-Cre; *n* = 10, LV-EGFP-Empty) is shown in ***C*** (preference for saccharin) and ***D*** (total fluid intake (g/kg/24 h). Each point is the average of 2 d of drinking ± SEM.

**Figure 7.**
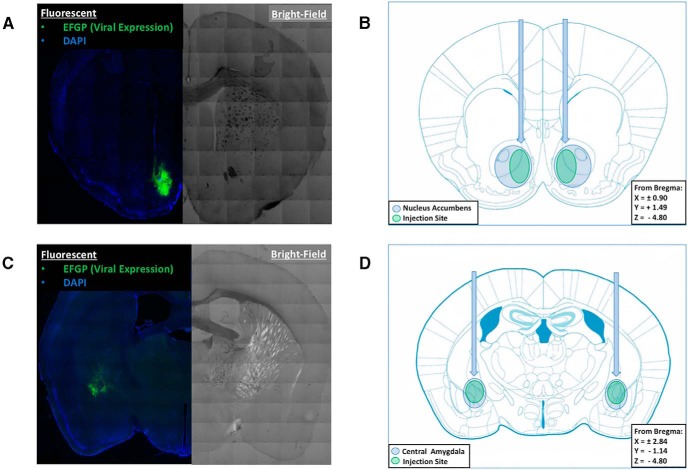
Injection target verification of lentiviral-mediated IKKβ knockdown in the NAc and CeA. ***A***, ***C***, Composite microscope images of a coronal section in the NAc (***A***) or CeA (***C***) of a representative lentiviral injection using fluorescent microscopy (on left) to show EGFP marker signal (green) and bright-field (on right) to demonstrate neuroanatomy. ***B***, ***D***, Coronal brain atlas figures of the injection sites with blue circles indicating the NAc (***B***) or CeA (***D***), and the green ovals illustrating the typical lentiviral injection location and spread.

Because ethanol drinking behavior in the 2BC test depends partly on taste (Bachmanov et al., 2003), we investigated the effect of the lentiviral-mediated knockdown of IKKβ in the NAc and CeA on preference for sweet/noncaloric (saccharin) solutions. After the ethanol drinking experiments, we administered a 2BC test using three different concentrations of saccharin versus water. Analysis of preference for saccharin indicated a significant main effect of concentration in both the NAc (*F*_(2,56)_ = 69.97, *p* < 0.0001) and CeA (*F*_(2,56)_ = 53.43, *p* < 0.0001), but no effect of treatment (LV-EGFP-Cre, LV-EGFP-Empty) or treatment × concentration interaction (Figs. 6*A*, 7*C* , respectively). Analysis of total fluid intake revealed no significant differences between the LV-EGFP-Cre and LV-EGFP treatment groups (Figs. 6*B*, 7*D* ). Thus, the knockdown of IKKβ in either the NAc or CeA did not change the preference for saccharin.

Upon completion of the behavioral experiments (∼8 weeks post-injection), the knockdown of IKKβ in the NAc and CeA was verified by (1) anatomical assessment of needle placement and viral spread, (2) confirmation of IKKβ protein knockdown, and (3) exploration of changes in mRNA levels of *Ikkb* and downstream proinflammatory cytokines in the NF-κB canonical pathway. To assess needle placement and viral spread, animals were perfused and brains harvested from a subset of the lentiviral-treated *Ikkb^F/F^* mice used in the brain region-specific IKKβ knock-down experiments (NAc: *n* = 22, LV-EGFP-Cre; *n* = 14, LV-EGFP-Empty; CeA: *n* = 15, LV-EGFP-Cre; *n* = 5, LV-EGFP-Empty). Injection coordinates and coverage of the NAc and CeA were verified using immunofluorescence to detect EGFP. Figure 7, *A* and *C*, shows representative images of coronal sections in the NAc (AP +1.49 mm) and CeA (AP −1.14 mm), respectively, of the *Ikkb^F/F^* mice treated with either LV-EGFP-Cre or LV-EGFP-Empty. The left side of the fluorescent image shows the EGFP signal (surrogate marker for lentiviral transduction) in green and DAPI (a stain that visualizes the nuclei of all cells) in blue. The right side of the image is a bright-field image used to better visualize the neuroanatomical landmarks. Figure 7, *C* and *D*, shows coronal sections from a mouse brain atlas in the area of the desired target coordinates with the blue circles showing the NAc and CeA, and the green ovals demonstrating the typical area where the LV-EGFP-Cre and LV-EGFP-Empty treatments were expressed. After completion of the drinking tests, the analysis of brain sections from knockdowns in NAc and CeA revealed that 100% of the samples met the criteria of (1) needle placement in at least one side within ±0.3 mm of the desired stereotaxic coordinates and (2) viral expression coverage that was greater than one-third of the area in the brain region of interest. The average viral coverage per injection site as indicated by the EGFP signal was 37.8% ± 4.8 in the NAc and 50.9% ± 5.7 in the CeA (mean ± SEM).

**Figure 8.**
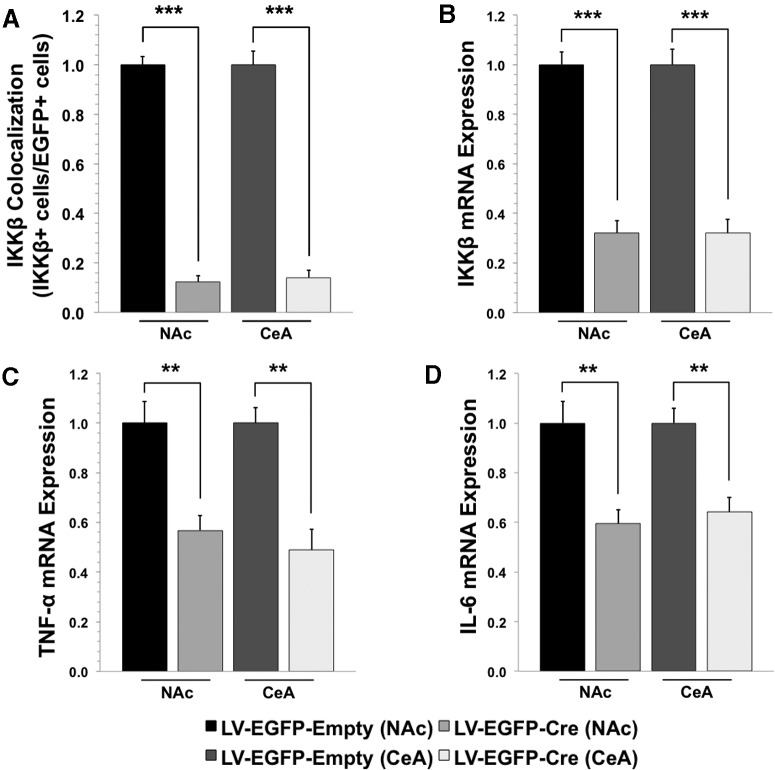
IKKβ protein levels and mRNA expression of IKKβ, TNF-α, and IL-6 at the injection site upon completion of behavioral studies. ***A***, IKKβ protein levels in NAc and CeA (***A***; *n* = 5 per group: NAc LV-EGFP-Cre, NAc LV-EGFP-Empty, CeA LV-EGFP-Cre, and CeA LV-EGFP-Empty). ***B–D***, mRNA levels of IKKβ (***B***), TNF-α (***C***), and IL-6 (***D***) in the NAc (*n* = 10, LV-EGFP-Cre; *n* = 5, LV-EGFP-Empty) and CeA (*n* = 5, LV-EGFP-Cre; *n* = 5, LV-EGFP-Empty). Values are shown relative to LV-EGFP-Empty-treated mice. IKKβ protein levels were analyzed using immunohistochemistry. IKKβ mRNA levels at the target site in the NAc and CeA were assessed by quantitative RT-PCR and normalized relative to GADPH. **p < 0.01, ***p < 0.001 determined by Student’s *t* test. All data are shown as the mean ± SEM.

After the 2BC drinking tests, IKKβ protein knockdown was confirmed in a subset of mice from the NAc and CeA experiments using immunohistochemistry (*n* = 5, LV-EGFP-Cre; *n* = 5, LV-EGFP-Empty). Brains were prepared, immunostained, and analyzed in the same manner as the IKKβ knock-down experiment (after 3 and 8 weeks) previously described. The relative expression of IKKβ in Cre-treated animals versus control was 0.122 ± 0.026 (*p* < 0.001) in the NAc and 0.141 ± 0.028 (*p* < 0.001) in the CeA (mean ± SEM; Fig. 8*A*). These represent an 88% and 86% decrease, respectively, in the NAc and CeA. These results were consistent with those obtained in pilot IKKβ knock-down experiments 8 weeks post-injection.

To determine changes in mRNA levels of *Ikkb* and downstream cytokines in the NF-κB canonical pathway, we performed quantitative PCR on micropunches from the NAc and CeA. A subset of slices from NAc (*n* = 10, LV-EGFP-Cre; *n* = 6, LV-EGFP-Empty) and CeA (*n* = 5, LV-EGFP-Cre; *n* = 5, LV-EGFP-Empty) experiments were harvested, flash frozen, sectioned, and micropunches were collected at the injection site. The relative expression of *Ikkb* was 0.321 ± 0.049 (*p* < 0.001) in the NAc and 0.360 ± 0.056 (*p* < 0.001) in the CeA; *Tnf* expression was 0.568 ± 0.059 (*p* < 0.01) in the NAc and 0.488 ± 0.084 (*p* < 0.01) in the CeA; and *Il6* expression was 0.595 ± 0.055 (*p* < 0.01) in the NAc and 0.641 ± 0.060 (*p* < 0.01) in the CeA (mean ± SEM). These values indicate ∼68% and 64% decrease in *Ikkb* mRNA in the NAc and CeA, respectively, and ≥35% knockdown of *Tnf* and *Il6* mRNA in both brain regions (Fig. 8).

### IKKβ was expressed primarily in neurons in the NAc and CeA

**Figure 9.**
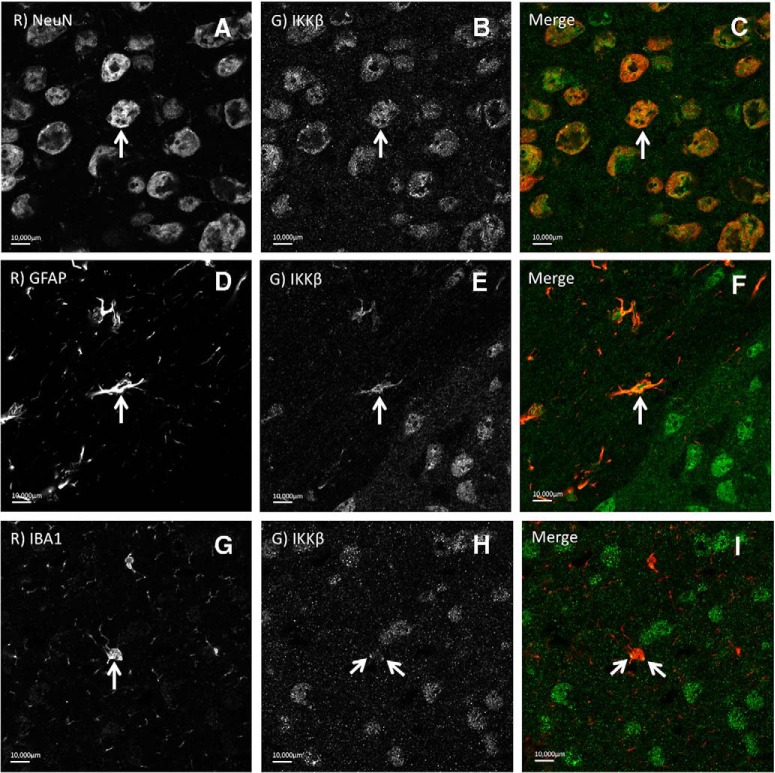
Cell type-specific localization of IKKβ in the NAc and CeA. ***A–I***, Representative fluorescent light microscope images illustrating cell type-specific antibodies in the first columns [anti-NeuN for neurons (***A***); anti-GFAP for astrocytes (***D***); anti-IBA1 for microglia (***G***)], anti-IKKβ stains in the second columns (***B***, ***E***, ***H***), and overlay of the first two in the third columns (***C***, ***F***, ***I***). Arrows illustrate cells showing colocalization of anti-IKKβ and cell type-specific stains.

To further investigate the specificity of IKKβ in these regions, we determined the cell-type localization of IKKβ in the NAc and CeA. Brain slices were costained using antibodies against IKKβ, and three common cell-type markers in the brain (neurons, anti-NeuN; astrocytes, anti-GFAP; microglia, anti-IBA1) from three adult male alcohol-naive C57BL/6J mice. Using fluorescent light microcopy to visualize IKKβ signal colocalization, we observed that in both the NAc and CeA, IKKβ was expressed in all three cell types to some degree, but was primarily expressed in neurons (Fig. 9).

**Figure 10.**
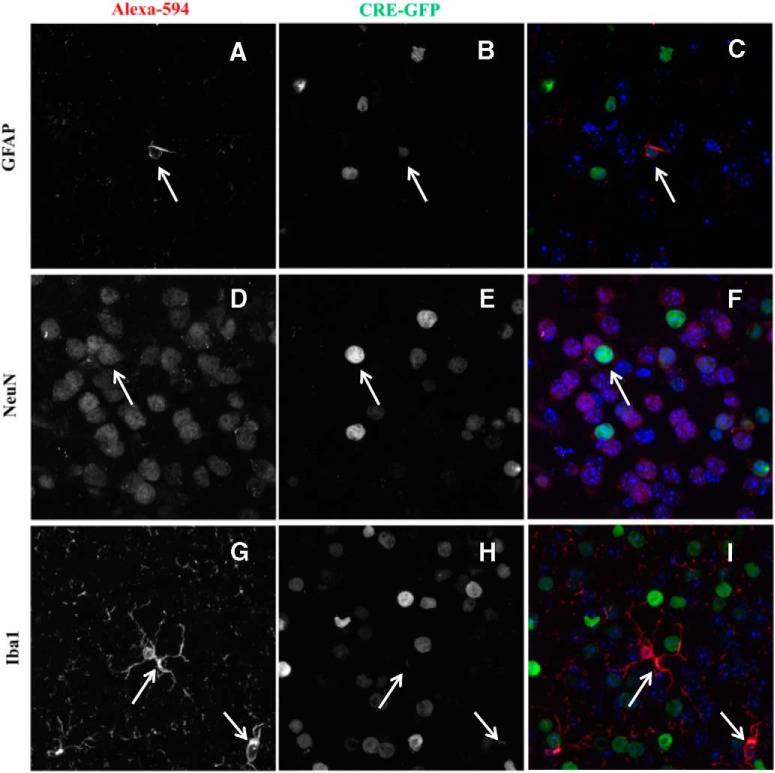
Cell-type trophism of lentiviral vectors in the NAc and CeA. ***A–I***, Representative fluorescent light microscope images illustrating cell type-specific stains in the first columns [anti-GFAP for astrocytes (***A***); anti-NeuN for neurons (***D***); anti-Iba1 for microglia (***G***)], anti-GFP stains in the second columns (***B***, ***E***, ***H***), and overlay of the first two in the third columns (***C***, ***F***, ***I***). Arrows illustrate cells showing coexpression of anti-GFP and cell type-specific stains.

Subsequently, we examined the trophism of the viral vector delivery system by costaining brain slices from LV-EGFP-Cre-treated animals in the NAc and CeA (*n* = 2, NAc LV-EGFP-Cre; *n* = 2, CeA LV-EGFP-Cre) using an antibody to target EGFP and the same three cell-specific markers described above. EGFP under the control of a CMV promoter in the VSV-G pseudotyped lentiviral vectors was expressed primarily in neurons (74.6 ± 1.3%), slightly in astrocytes (10.8 ± 2.2%), and only marginally in microglia (1.8 ± 0.5%; Fig. 10).

### Identification of NF-κB targets

We previously examined chronic ethanol-induced changes in gene expression patterns in mouse brain (Osterndorff-Kahanek et al., 2015) and used these datasets to determine changes in downstream *Nfkb/Rel* gene targets in mouse CeA, NAc, and PFC. Ingenuity pathway analysis (IPA) was used to curate and identify potential gene targets in mice and humans. As shown in Table 1, we identified numerous targets, suggesting that large networks of downstream genes may be altered by ethanol. Ethanol has also been shown to alter gene expression of IKKβ in mice (Osterndorff-Kahanek et al., 2015) and humans (Flatscher-Bader et al., 2005; Mayfield et al., unpublished observations), and IKKβ may represent an upstream intermediate target to control NF-κB activation and reduce alcohol-induced changes in gene expression.

**Table 1:** Ethanol-induced changes in NFKB/REL gene targets in mouse brain

CeA		NAc		PFC	
Illumina probe ID	NFKB/REL targets (IPA)	Illumina probe ID	NFKB/REL targets (IPA)	Illumina probe ID	NFKB/REL targets (IPA)
ILMN_2738825	*ACTA1*	ILMN_2878060	*ANXA6*	ILMN_2878060	*ANXA6*
ILMN_2739999	*B2M*	ILMN_2739999	*B2M*	ILMN_1221503	*CCND1*
ILMN_1216746	*B2M*	ILMN_1216746	*B2M*	ILMN_2601471	*CCND1*
ILMN_2717613	*CDK2*	ILMN_2706514	*BCL2*	ILMN_2931411	*CCT3*
ILMN_2756435	*CEBPB*	ILMN_2716567	*BNIP3L*	ILMN_2846775	*CDKN1A*
ILMN_2609813	*CHI3L1*	ILMN_2756435	*CEBPB*	ILMN_2634083	*CDKN1A*
ILMN_1224754	*CKB*	ILMN_2959480	*EIF4A1*	ILMN_2846776	*CDKN1A*
ILMN_2747651	*HAT1*	ILMN_2718815	*FAF1*	ILMN_2992403	*CINP*
ILMN_2624153	*HES5*	ILMN_2994806	*H2AFJ*	ILMN_2627041	*CX3CL1*
ILMN_1253414	*HES5*	ILMN_2648292	*H3F3A/H3F3B*	ILMN_1256348	*DNTTIP1*
ILMN_2791952	*HES6*	ILMN_1253414	*HES5*	ILMN_2737713	*EDN1*
ILMN_1222313	*HIST1H4J*	ILMN_2791952	*HES6*	ILMN_2903945	*GADD45G*
ILMN_2924419	*HLA-A*	ILMN_2855315	*HIST1H1C*	ILMN_2791952	*HES6*
ILMN_2835683	*HLA-A*	ILMN_2924419	*HLA-A*	ILMN_2835683	*HLA-A*
ILMN_1235470	*HNRNPC*	ILMN_2835683	*HLA-A*	ILMN_3156604	*HMGB2*
ILMN_1239021	*HNRNPM*	ILMN_2715802	*HMGA1*	ILMN_2664319	*IRF3*
ILMN_2646625	*JUN*	ILMN_1235470	*HNRNPC*	ILMN_2646625	*JUN*
ILMN_2878071	*LYZ*	ILMN_2511051	*HNRNPC*	ILMN_3001914	*NFKBIA*
ILMN_2640883	*NDE1*	ILMN_2921103	*HNRNPM*	ILMN_2596979	*NRARP*
ILMN_2596979	*NRARP*	ILMN_2921095	*HNRNPM*	ILMN_3158919	*PRKCZ*
ILMN_1242466	*PSMB9*	ILMN_1239021	*HNRNPM*	ILMN_2647533	*SLC41A3*
ILMN_2717621	*RPS15A*	ILMN_2646625	*JUN*	ILMN_2938893	*SMAD3*
ILMN_2883164	*SERPINE2*	ILMN_1258376	*KCNK6*	ILMN_2701664	*TSC22D3*
ILMN_2701664	*TSC22D3*	ILMN_1258526	*LGALS3BP*	ILMN_3150811	*TSC22D3*
ILMN_3150811	*TSC22D3*	ILMN_2878071	*LYZ*	ILMN_3112873	*TXNIP*
ILMN_3121255	*VEGFA*	ILMN_2640883	*NDE1*		
		ILMN_2596979	*NRARP*		
		ILMN_2790181	*PHGDH*		
		ILMN_1234766	*PPME1*		
		ILMN_2833378	*PRKACA*		
		ILMN_1242466	*PSMB9*		
		ILMN_1248316	*PTGDS*		
		ILMN_2728729	*SDC4*		
		ILMN_3027751	*SORBS1*		
		ILMN_2988299	*SRF*		
		ILMN_2692615	*TGM2*		
		ILMN_2701664	*TSC22D3*		
		ILMN_3150811	*TSC22D3*		
		ILMN_3112873	*TXNIP*		

Ethanol administration produced numerous changes in NFKB/REL gene targets in mouse CeA, NAc, and PFC. IPA was used to curate gene targets. Gene target identification included both human and mouse databases. Human nomenclature is used here. Adjusted *p* values are listed using an FDR of *p* < 0.001. Experimental details are provided in the study by Osterndorff-Kahanek et al. (2015).

## Discussion

IKKβ is a critical component in the regulation of the NF-κB inflammatory cascade, but its role in alcohol drinking had not been investigated prior to this study.

Inhibiting IKKβ, either peripherally or in brain regions associated with addictive behaviors, decreased voluntary ethanol consumption and preference in several drinking tests, including long-term and binge-like paradigms. Systemic administration of the peripherally acting IKKβ inhibitors, TPCA-1 or sulfasalazine, reduced ethanol drinking in two distinct drinking models (2BC and 2BC-DID). The ability of sulfasalazine and TPCA-1 to penetrate the BBB is not well established, and their anti-inflammatory effects are thought to be confined to the periphery (Liu et al., 2012). However, it is possible that the inhibition of systemic inflammatory signaling during drinking also impacts central pathways. Decreased levels of proinflammatory cytokines in blood, for example, could translate to decreased cytokine release and signaling across the BBB, ultimately decreasing levels of inflammatory mediators in brain. We also note that other anti-inflammatory agents, such as minocycline, were proposed to reduce drinking in mice through direct central actions (Agrawal et al., 2014). It has been hypothesized that alcohol-induced inflammatory responses signal via peripheral-central positive-feedback cycles (Robinson et al., 2014). Regardless of the primary mechanism, the ability of peripheral IKKβ inhibitors to successfully inhibit long-term and binge-like drinking alludes to their translational potential as a therapeutic target.

Knockdown of IKKβ in the NAc or CeA was sufficient to decrease voluntary 2BC ethanol consumption, showing that drinking behavior can be selectively regulated by the central actions of IKKβ. The NAc is part of the mesolimbic dopamine reward system, which has a well documented role in substance abuse, and has also been implicated in the rewarding effects of cocaine mediated by IKKβ (Russo et al., 2009). The CeA is involved in fear-motivated behaviors associated with drug and alcohol abuse, and has been shown to mediate the behavioral effects of ethanol consumption in rodents (Roberto et al., 2004a,b; 2006; Lam et al., 2008). Lesions of the central, but not basolateral, amygdala decreased voluntary ethanol consumption (Möller et al., 1997), and a review of the neurocircuitry of drug addiction further highlights the role of plasticity in frontal cortical and subregions of the amygdala in craving, withdrawal, negative affect, and loss of control (Koob and Volkow, 2010). Thus, the brain regions targeted here have key roles in alcohol addiction-mediated behaviors and were both sensitive to IKKβ knockdown.

We provide initial evidence that IKKβ knockdown disrupts proinflammatory cascades in the NAc and CeA based on decreased expression of downstream products of the NF-κB canonical pathway (TNF-α and IL-6) in both regions. Although the corresponding reductions in these inflammatory cytokines suggest that this pathway is responsible for the decreased drinking, these results do not provide specific mechanistic evidence of downstream effects. Other studies (Blednov et al., 2011; Robinson et al., 2014) have hypothesized that alcohol-induced increases in levels of cytokines promote excessive alcohol consumption in animal models and human alcoholic subjects. This may in turn exacerbate inflammatory responses via activation of NF-κB. In fact, NF-κB DNA binding in the brain has been shown to increase with ethanol treatment (Crews et al., 2006) and the human *NFKB1* gene has also been linked with alcoholism (Edenberg et al., 2008).

In addition, previous evidence suggests that long-term ethanol consumption alters gene expression of IKKβ in mouse PFC (Osterndorff-Kahanek et al., 2015) and human postmortem PFC from alcoholic subjects (Flatscher-Bader et al., 2005). Furthermore, our current evidence suggests that NF-κB-related gene targets are ethanol-responsive in mouse CeA, NAc, and PFC, potentially affecting a significant number of downstream targets (mouse and human). Based on the genomic evidence in mice and humans, and the many networks of downstream genes that may be dysregulated, ethanol-mediated changes in IKKβ regulation of NF-κB cascades are relevant targets that may offer new treatment strategies for AUD.

In addition to different brain regions, different cell types may play unique roles in inflammatory responses (Szabo and Lippai, 2014; Lacagnina et al., 2016; Warden et al., in press). Inflammatory pathways are not limited to glia or other immunocompetent cells, but also involve neurons and neuronal–glial interactions. In our study, the selective knockdown of IKKβ did not affect all cell types equally, due in part to the viral delivery system. IKKβ was expressed primarily in neurons in the NAc and CeA with lesser amounts found in glia (e.g., astrocytes and microglia). The cell-type specificity of the viral vector system delivering *Cre* favored the transduction in neurons, and to a lesser degree in astrocytes, and only marginally in microglia. Even though IKKβ was knocked down to some extent in all three cell types, neurons appear to be a primary target. Because GFAP only labels a subset of astrocytes in the CNS and is not an ideal marker, our estimation that IKKβ knockdown occurred in only 10% of astrocytes may be an underestimation. While this caveat warrants the consideration of the role of IKKβ in astrocytes, it does not detract from the novel evidence that neurons are involved in the IKKβ-mediated reduction in ethanol drinking.

The IKK complex (IKKα, IKKβ, IKKγ/NEMO) is a crucial mediator for several proinflammatory pathways that ultimately result in the activation of NF-κB. IKKβ primarily regulates the NF-κB canonical pathway (transcription of inflammatory genes/antiapoptosis), IKKα regulates the NF-κB noncanonical pathway (cell cycle regulation/proliferation), while IKKγ/NEMO participates in both pathways (Perkins, 2007; Gamble et al., 2012). We suggest that the knockdown of IKKβ in the NAc and CeA targeted the canonical pathway in neurons and, to some extent, astrocytes, interrupting inflammatory signaling and feedback cycles.

The central effects of IKKβ are not well known, and prior to this work its role in alcohol drinking had not been investigated. Our results provide novel evidence that peripheral and/or central inhibition of IKKβ decreases ethanol drinking, including binge-like consumption. Ethanol could induce peripheral cytokines that ultimately activate expression of immune-related genes in the brain or could directly stimulate central immune- and inflammatory-related pathways. Inhibiting IKKβ-mediated signaling could dampen the peripheral as well as the central inflammatory effects of ethanol. Our results are consistent with other studies showing that null mutant mice lacking genes associated with proinflammatory pathways had reduced levels of chemokines and cytokines, and reduced voluntary ethanol consumption (Blednov et al., 2005, 2012). However, not all inflammatory-related genes studied to date have been shown to regulate ethanol drinking in mouse knock-out models (Mayfield et al., 2016), suggesting that indiscriminant inhibition of inflammatory pathways is not a viable strategy to limit excessive drinking and further highlighting the relevance of the current study in targeting treatment strategies.

In summary, voluntary ethanol drinking was decreased by inhibiting IKKβ peripherally using pharmacological inhibitors or centrally using genetic deletions in the CeA or NAc, regions known to be important in the neurobiology of alcohol abuse (Koob and Volkow, 2010). Although the effects of inflammatory pathways are often attributed to glia (astrocytes and microglia), this study highlights a novel neuronal role for IKKβ in alcohol consumption. Our results also provide evidence that the use of peripherally acting IKKβ inhibitors with anti-inflammatory properties is a potential treatment strategy for decreasing alcohol drinking. In particular, drugs like sulfasalazine, which are already approved by the Food and Drug Administration, may provide fast-track treatment options for AUD or other inflammatory-related diseases. Studies such as this that probe key inflammatory pathways, peripheral–central components, different drinking models, and brain-region and cell-type specificity will continue to refine treatment strategies and opportunities for AUD.

## Synthesis

The decision was a result of the Reviewing Editor Karen Szumlinski and the peer reviewers coming together and discussing their recommendations until a consensus was reached. A fact-based synthesis statement explaining their decision and outlining what is needed to prepare a revision is listed below.

I have read the 2nd revision of the manuscript authored by Truitt et al, which was originally submitted to J Neurosci. While Reviewer #2 was completely satisfied with all of the revisions provided by the authors, Reviewer #1 persisted with concerns that I frankly considered ill-founded. In my opinion, this 2nd version of the manuscript is very well-written and wholly acceptable for publication in eNeuro. The appropriate controls are employed, the data presented in a very clear manner, the efficacy and localization of their shRNA approach is well-documented and the findings provide an important new lead in the neurobiology of alcoholism. As such, I have issued the decision of Accept As Is.
